# Use of SLAM and PVRL4 and Identification of Pro-HB-EGF as Cell Entry Receptors for Wild Type Phocine Distemper Virus

**DOI:** 10.1371/journal.pone.0106281

**Published:** 2014-08-29

**Authors:** Mary M. Melia, John Philip Earle, Haniah Abdullah, Katherine Reaney, Frederic Tangy, Sara Louise Cosby

**Affiliations:** 1 Centre for Infection and Immunity, School of Medicine, Dentistry and Biomedical Sciences, Queen's University Belfast, Belfast, United Kingdom; 2 Viral Genomics and Vaccination Laboratory, Institut Pasteur, CNRS-URA3015, Paris, France; University of Pennsylvania School of Veterinary Medicine, United States of America

## Abstract

Signalling lymphocyte activation molecule (SLAM) has been identified as an immune cell receptor for the morbilliviruses, measles (MV), canine distemper (CDV), rinderpest and peste des petits ruminants (PPRV) viruses, while CD46 is a receptor for vaccine strains of MV. More recently poliovirus like receptor 4 (PVRL4), also known as nectin 4, has been identified as a receptor for MV, CDV and PPRV on the basolateral surface of polarised epithelial cells. PVRL4 is also up-regulated by MV in human brain endothelial cells. Utilisation of PVRL4 as a receptor by phocine distemper virus (PDV) remains to be demonstrated as well as confirmation of use of SLAM. We have observed that unlike wild type (wt) MV or wtCDV, wtPDV strains replicate in African green monkey kidney Vero cells without prior adaptation, suggesting the use of a further receptor. We therefore examined candidate molecules, glycosaminoglycans (GAG) and the tetraspan proteins, integrin β and the membrane bound form of heparin binding epithelial growth factor (proHB-EGF),for receptor usage by wtPDV in Vero cells. We show that wtPDV replicates in Chinese hamster ovary (CHO) cells expressing SLAM and PVRL4. Similar wtPDV titres are produced in Vero and VeroSLAM cells but more limited fusion occurs in the latter. Infection of Vero cells was not inhibited by anti-CD46 antibody. Removal/disruption of GAG decreased fusion but not the titre of virus. Treatment with anti-integrin β antibody increased rather than decreased infection of Vero cells by wtPDV. However, infection was inhibited by antibody to HB-EGF and the virus replicated in CHO-proHB-EGF cells, indicating use of this molecule as a receptor. Common use of SLAM and PVRL4 by morbilliviruses increases the possibility of cross-species infection. Lack of a requirement for wtPDV adaptation to Vero cells raises the possibility of usage of proHB-EGF as a receptor in vivo but requires further investigation.

## Introduction

Morbilliviruses constitute a genus within the family *Paramyxoviridae*, subfamily *Paramyxovirinae*. Members of the genus include measles virus (MV), canine distemper virus (CDV), rinderpest virus (RPV), peste des petits ruminants virus (PPRV) and the more recently identified marine morbilliviruses, phocine distemper virus (PDV), dolphin morbillivirus (DMV) and porpoise morbillivirus (PMV). DMV and PMV are now considered a single species of cetacean virus. Due to the close sequence similarity and cross-species infection PDV is thought to be derived from CDV through contact between terrestrial carnivores and seals [1].

The expression of specific viral receptors on the host cell surface is a prerequisite for virus entry and is a major determinant of tissue tropism and viral pathogenesis. There is a concern that once MV is eradicated and vaccination discontinued that one or more of the veterinary morbillivirus could cross species into humans as is thought to have occurred in the past. Phylogenetic evidence suggests that MV may have evolved from RPV following domestication of cattle [2]. There is a new threat as in recent years CDV has caused outbreaks in non-human primates [3]–[5]. Studies in dogs and non-human primates indicate that continuing vaccination after eradication of MV is likely to provide a sufficient degree of cross-protection to protect humans against other morbilliviruses [6], [7]. However, it is important to determine the use of common or unique cell entry receptors across the morbillivirus genus as an indicator of cross-species infection potential.

The hemagglutinin (H) and fusion (F) proteins of morbilliviruses are responsible for attachment to and fusion with the host cell plasma membrane, respectively and therefore the expression of both proteins is necessary for efficient cell entry [8]. Morbilliviruses infect a number of different cell types in vivo including leucocytes, epithelial, endothelial and neural cells with the need for more than one receptor type [9].

Signalling lymphocyte activation molecule (SLAM) or CD150 is a member of the C2 subset of the immunoglobulin superfamily and is expressed on activated B and T cells, constitutively on immature thymocytes, memory T cells, a proportion of B cells as well as activated monocytes and mature dendritic cells [10]–[12]. SLAM has been identified as a receptor for both vaccine and wild type (wt) strains of MV as well as for CDV, RPV and PPRV [13]–[16]. The immune cell types infected are predominantly T, B and dendritic cells [17]–[19] and morbilliviruses have been found to use SLAM of non-host species but with lower efficiency [14]. Vero cells expressing canine SLAM have been used to isolate wtPDV [20], [21] as it has been assumed that PDV uses this receptor but this has not been confirmed.

Molecules other than SLAM must mediate virus cell entry in other cell types such as epithelial, endothelial and neural cells. More recently PVRL4 (also known as nectin 4) has been identified as a receptor for MV [22], [23], CDV [24] and PPRV [16] on the basal surface of epithelial cells. We have also shown that PVRL4 is up-regulated in human brain endothelial cells by MV infection [25]. It is not known if PDV also uses this receptor.

CD46 (membrane cofactor protein), a type 1 transmembrane glycoprotein and a complement regulatory protein, found on all nucleated cells, was first identified as a MV receptor [26], [27]; however, it was later shown that most wtMV strains did not interact with CD46 [28], [29]. CD46 is not a receptor for CDV and RPV [30], [31]. However, there is evidence to suggest that CDV-H protein interacts with an unknown cellular receptor(s) regulated by CD9, a member of the tetraspan transmembrane- protein family [32]. Most tetraspans including CD9, associate with β1 integrins in the cell membrane [33]. Furthermore, CD46 has been shown to form a complex with CD9, beta1 integrins and the membrane bound form of heparin-binding EGF-like growth factor (pro-HB-EGF) [34], [35]. HB-EGF and proHB-EGF have a highly conserved heparin binding domain which is also the site of CD9 binding [36]. Therefore, investigation of both beta1 integrins and pro-HB-EGF as possible receptor molecules is of interest.

Other receptors for MV may act at the attachment stage but do not allow virus entry. The C-type lectin DC-specific intercellular adhesion molecule 3-grabbing nonintegrin DC-SIGN has been shown to be an attachment receptor to enhance CD46/CD150-mediated infection of Dendritic cells. The ligands for DC-SIGN are both MV glycoproteins F and H in contrast to CD46, SLAM and PVRL4 where only H is required [37]. In addition, human primary Langerin cells (a subset of DCs) are capable of capturing MV through the C-type lectin Langerin [38]. Glycosaminoglycans (GAG) have been shown to have a role in infection by a tissue culture-adapted vaccine strain of RPV and recombinant wt strains of CDV as well as MV [15]; [39], [40]. It has been suggested that GAG which are ubiquitously expressed may not cause viral entry but may support the binding of virus to a low affinity or low quantity receptor [40].

In this study we investigated the use of CD46, SLAM and PVRL4 as receptors for wtPDV and examined the interaction of selected morbilliviruses with other candidate molecules, β1 integrin, pro-HB-EGF and GAG.

## Materials and Methods

### Cell lines and viruses

Vero-dogSLAM (VDS) cells were obtained from R. Cattaneo, (Mayo Clinic, USA); B95a (an Epstein Barr virus-transformed marmoset B cell line), Chinese Hamster Ovary (CHO), CHO-CD46 and CHO-marmosetSLAM (CHO-MSLAM) cell lines from J. Schneider-Schaulies (University of Würzburg, Germany); CHO-dogSLAM (CHO-DSLAM) cells from V. von Messeling (Mayo Clinic, USA); CHO-humanPVRL4 cells, which lack heparin and chondroitin sulfate on their surface, from C. Richardson (Dalhousie University, Halifax, Canada). CHO empty plasmid (CHO-E) and CHO-human proHB-EGF cell lines were obtained from R. Adam (Harvard University, USA). Vero and VDS cells were grown in Dulbecco's Modified Eagles Medium containing 4500 mg/L glucose supplemented with 5% foetal calf serum (FCS). VDS cells had 1 mg/ml zeocin (Invitrogen, USA) added for selection of plasmid containing cells. B95a cells were grown in RPMI-1640 medium supplemented with 10% FCS. CHO, CHO-CD46 and CHO-SLAM cell lines were grown in Dulbecco's Modified Eagles Medium containing 4500 mg/L glucose supplemented with 5% FCS and 1 mg/ml zeocin (Invitrogen).CHO-PVRL4, CHO-proHB-EGF, CHO-E cells were grown as for the other CHO cell lines except 1 mg/ml geneticin (Invitrogen, USA) was used instead of zeocin for selection of plasmid containing cells. Cells were incubated at 37°C with 5% (v/v) CO_2_. The origin of cell lines and the known morbillivirus receptors on each of these are shown in Table 1.

**Table 1:** Cell lines and Known Receptors for Morbilliviruses.

Cell	Type	Known Morbillivirus Receptors
Vero	African Green Monkey Kidney	CD46 (vaccine strains of MV, not wtMV or CDV)
Vero-dogSLAM (VDS)	Vero-stably transfected with canine SLAM	CD46, SLAM
B95a	Adherent Marmoset B cell –Epstein Barr virus-transformed	SLAM (MV, CDV, RPV), non-functional CD46
CHO	Chinese hamster ovary	None
CHO-CD46	CHO stably transfected wth human CD46	MV vaccine strains
CHO-dogSLAM	CHO-stably transfected with canine SLAM	SLAM (MV,CDV, RPV)
CHO-MSLAM	CHO-stably transfected with marmoset SLAM	SLAM (MV, CDV, RPV)
CHO-PVRL4	CHO-stably transfected with human PVRL-4	PVRL4 (MV)
CHO-proHB-EGF	CHO-stably transfected with human proHB-EGF	Unknown
CHO-Empty	Stably transfected with empty plasmid	None

WtPDV isolates PDV/NL88n and PDV/USA2006 [21], MV Schwarz GFP [41], wtMV Dublin-3267 strain [42], the Edmonston strain of MV, the Snyder Hill wtCDV strain (obtained from M. Appel, Cornell University, USA) and the Onderstepoort strain of CDV were grown and titred by TCID_50_ in VDS cells. The origin and cell passage history of the viruses are shown in Table 2. Unless otherwise stated all infections were carried out at a multiplicity of infection (MOI) of 0.1.

**Table 2:** Virus Origin and Isolation.

Virus	Origin	Cell line of Isolation and growth
wtPDV/NL88n	Isolated in 2003 from blood of seal from 1988 Netherlands outbreak	B95a
wtPDV/USA2006	Isolated from seal, east coast of USA 2006	VDS
wtCDV Snyder Hill	Isolated from dog, Ithaca, NY, USA- early 1970's	Isolated in dog lung macrophages and 2 passages in VDS cells
MV Edmonston	Measles vaccine strain	Continual passage in Vero cells
MV Schwarz GFP	Measles recombinant vaccine strain expressing GFP	Continual passage in Vero cells
CDV Onderstepoot	CDV vaccine strain	Continual passage in Vero cells

### Growth Curves

Triplicate cultures in 24 well trays were infected at an MOI of 0.1 for 1 hour. Cultures were washed 6 times to remove non adsorbed virus and replaced with 1 ml of fresh maintenance medium supplemented with 2% foetal calf serum (FCS). Total virus was harvested from each well at selected time points and titrated in VDS cells.

### Sodium chlorate treatment

Cultures were grown for 48 h in 30 or 60 mM sodium chlorate. The medium was removed and cells were washed twice in PBS, infected as previously described and incubated with or without the respective concentration of sodium chlorate.

### Heparinase treatment

Vero cells were washed twice with PBS prior to incubation with 10 U/ml of Heparinase 1 (Sigma) and incubation at 37°C for 90 min. Cells were washed twice before infection. as previously described.

### Immunofluorescence

Cover slip cultures were fixed in 4% paraformaldehyde. Where intracellular staining for virus was required cells were permeabilised with Triton-X 100 (Sigma) and fixed before staining. For receptors, cells were not permeabilised and fixation was carried out after staining. Cells were initially incubated with blocking solution (0.5% bovine serum albumin in PBS). Following removal of blocking solution, cover slips were incubated for 1 hr at 37°C with appropriate primary antibodies or control serum diluted in blocking solution: Serum obtained from a patient with subacute scelerosing panencephalitis (SSPE, 1/1000 dilution) hyperimmune for MV and cross reactive with other morbilliviruses; Mouse anti-SLAM (5C6) anti-CD46 (13/42) monoclonals, obtained from J. Schneider–Schaulies, (both antibodies diluted1/1000) or alternatively mouse anti-SLAM (IPO-3, Research diagnostics Inc, USA, 1/1000) or mouse anti-CD46 (Abcam, 1/500 dilution); goat anti HB-EGF (Abcam, 1/500). Mouse isotype IgG1 (Abcam, 1/100) or normal goat serum Abcam, I/500) were used as appropriate controls. All secondary antibodies were diluted in PBS and cover slips washed and incubated for 1 hr at 37°C with the appropriate FITC-conjugate: rabbit anti-human IgG (Abcam, 1/50); rabbit anti-mouse (DAKO A/S, Denmark, I/100) or donkey anti-goat (Abcam, 1/100). The coverslips were then mounted in vectashield mounting medium with DAPI (Vector Laboratories) or propidium iodide (Abcam). In each experiment controls consisting of non-infected cells and coverslips treated with appropriate control serum instead of primary antibody were included. Images were examined using a BioRad Microradiance Confocal Olympus BX60 epifluorescent or a UV Eclipse TE2000-U (Nikon) microscope.

### Receptor blocking assay

Cells were treated with 10µg/ml of appropriate antibodies or control serum in the appropriate medium and incubated at 37°C for 1 h prior to the addition of virus at an MOI of 0.1. Further incubation was carried out for selected times in the presence of the antibody.

### Receptor Binding Assay

Monolayers were treated with virus at an MOI of 10 at 4°C and washed 3 times with PBS. RNA was extracted using TriZol reagent (Sigma). Sybr green qRT-PCR was carried out using a qRT-PCR system from Alignment Technology in a 7500 Teal Time PCR system (Applied Biosystems). The copy number for virus RNA was determined from a standard curve. MV P gene and β actin primers and the assay conditions were previously described [43]. The PDV Phosphoprotein (P) gene primers sequences used were:-

Forward 5′ CCA TTA AAA AGG GCA CAG GA

Reverse 5′ GTT TCT CGG GTT GGG GTC TCG TA

### Flow cytometry analysis

Staining for flow cytometry was carried out according to the BD Cytofix/Cytoperm Fixation/Permeablization Kit manufacturer's instructions (BD, UK). Cells were washed 3 times in PBS and 1×10^5^ cells suspended in 1/100 dilution of SSPE serum, incubated at 37°C for 1 h, washed and incubated with FITC-conjugated rabbit anti-human IgG (1/50 dilution) at 37°C for 1 h. Cells were washed and resuspended in PBS with 1% FCS. The samples were examined in a (PartecCyFlow Space) and analyzed by a FlowJo programme (Oregon, USA). Experiments were repeated at least three times.

### Statistical analysis

Statistical analysis was carried out by using the student T-test. Values of p<0.05 were considered statistically significant*,p<0.01 very significant ** and <0.001 highly significant ***.

## Results

### wtPDV infects Vero cells

**Figure 1:**
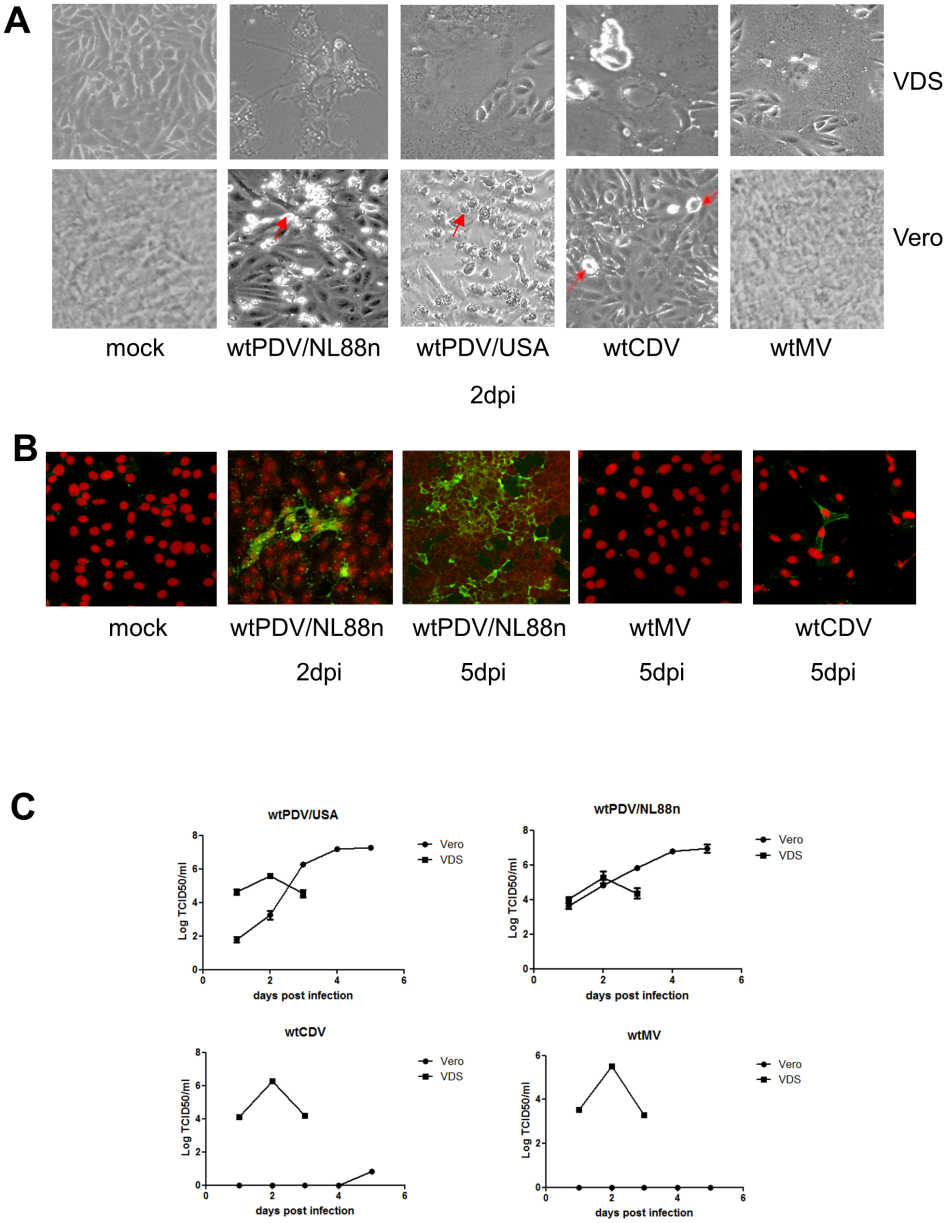
wtPDV infects Vero cells. Vero and VDS cells were infected at an MOI of 0.1. (A) CPE observed in Vero and VDS cultures infected with wtPDV/NL88n, wt PDV/USA2006, wtCDV and wtMV at 2 dpi by phase contrast microscopy (Magnification X100). Foci of rounded cells are indicated by arrows. (B) Cells were infected with wtPDV/NL88n, wtCDV and wtMV, fixed, permeabilised and stained with SSPE serum and rabbit anti-human FITC; nuclei were stained with propidium iodide. Images were taken using a Nikon Eclipse TE2000-U UV microscope (x400). (C) Vero cells and VDS cells were infected with wtPDV/NL88n, wtPDVUSA2006, wtMV and wtCDV for up to 5 days. Titres were determined by TCID_50_ in VDS cells. The results are representative of two independent experiments.

To determine if PDV uses SLAM as a receptor we infected Vero and VDS cells at an MOI of 0.1 with wtPDV/NL88n, wt PDV/USA2006, wtCDV and wtMV. Large syncytia formation was first observed in VDS cells infected with all viruses at 1 dpi with the monolayer completely fused by 2 days post infection (dpi) (Figure 1A top panel) and disintegration after 3 days. Also as expected wtMV failed to produce CPE in Vero cells while a few isolated rounded cells were noted in wtCDV infected cultures at 2 dpi. However, in contrast both wtPDV strains produced extensive cell rounding at 2 dpi (Figure 1A bottom panel). To verify that the cell rounding was caused by the virus, immunofluorescence staining of PDV/NL88n, wtCDV and wtMV inoculated cultures using SSPE antiserum (which cross-reacts with all morbilliviruses) was carried out. In wt PDV cultures the rounded cells observed were found to contain virus at 2 dpi with more extensive infection by 5 dpi when a limited amount of fusion occurred. A single foci of infection is shown for wtCDV at 5 days dpi while no antigen was detected in wtMV cultures (Figure 1B).

Further Vero and VDS cultures were infected at an MOI of 0.1 with wtPDV/NL88n, WTPDV/USA2006 wtMV and wtCDV and total virus yields (determined by titration on VDS cells) were compared from 1 to 5 dpi. The virus titres obtained were not significantly different at 2 dpi between Vero and VDS cells. Due to total destruction of the monolayers in VDS cells by 3dpi further titres could not be determined. In contrast, the titre in Vero cells substantially increased by 5 dpi indicating prolonged production of virus. WTMV and wtCDV strains showed no and minimal virus growth, respectively (Figure1C). Altogether, these results indicated that SLAM expression is dispensable for the entry of wtPDV in Vero cells.

### Infection of Vero Cells by wtPDV is not dependent on CD46

**Figure 2:**
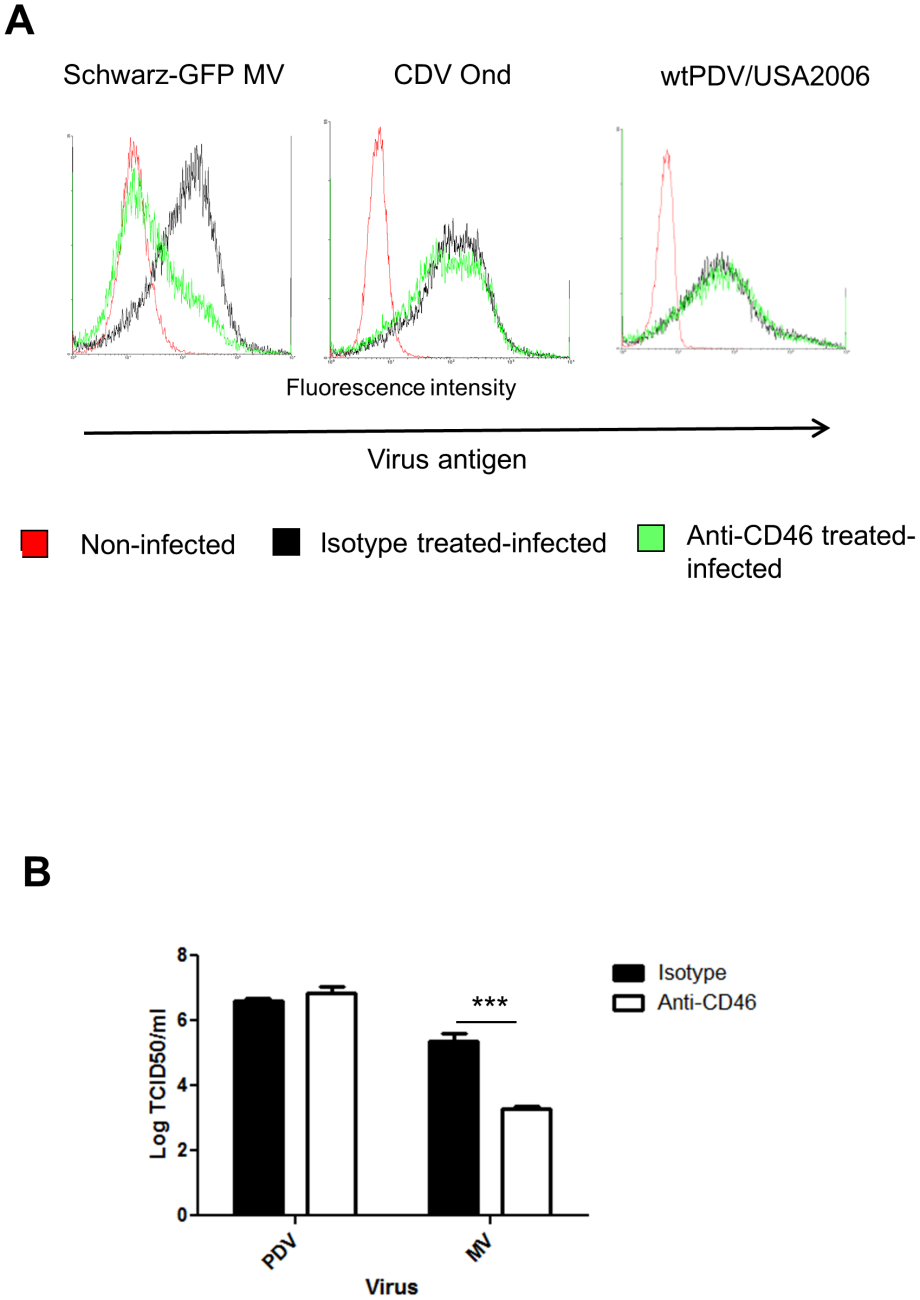
Infection of Vero cells by wtPDV is not dependent on CD46. Vero cells were incubated with anti-CD46 monoclonal antibody or mouse isotype control prior to virus infection (MOI 0.1) for 2 days (Schwarz-GFP MV and Onderstepoort CDV) or 5 days (wtPDV/USA2006). (A) Mock infected and infected cells were fixed before staining (with the exception of Schwarz-GFP MV) with SSPE serum followed by rabbit anti-human FITC and analysed by flow cytometry. (B) Virus was harvested from cells infected with wtMV and wtPDV and titres determined by TCID_50_ in VDS cells. The results are representative of two independent experiments.

As wtPDV could infect Vero cells in the absence of SLAM, we examined the use of CD46 as a receptor for this virus. Since wtMV and wtCDV strains do not infect Vero cells (Figure 1A and Figure 1C), a vaccine strain of MV, which uses CD46 as a receptor in Vero cells, and the Onderstepoort vaccine strain of CDV, which infects Vero cells in a CD46 independent manner were used as positive and negative controls, respectively. Vero cells were treated with anti-CD46 antibody for 1 h and infected at an MOI of 0.1 with wtPDV/USA2006 (for 5 days), Schwarz-GFP MV and CDV Onderstepoort (for 2 days) when the majority of cells are infected in isotype control treated cultures. Infected and mock infected cells were fixed, stained with SSPE serum (with the exception of Schwarz-GFP MV) and flow cytometry carried out for all three viruses. In parallel, virus was harvested from MV (at 2 days) and PDV (at 5 days) infected cultures and titres determined by TCID_50_. As expected GFP production was significantly reduced in cells treated with anti-CD46 antibody and infected with Schwarz-GFP MV. The single peak of fluorescence indicates that cells have a continuous range of GFP expression, the majority with no expression and those in the tail of the peak with levels equivalent to cells treated with the isotype serum control. Anti-CD46 antiserum had no effect on CDV or wtPDV antigen production (Figure 2A) while the MV virus titre was reduced by 2 logs (Figure 2B). Therefore, these results exclude the use of CD46 as a receptor for PDV.

### Infection of B95a cells with wtPDV is partially inhibited by anti-SLAM antibody

**Figure 3:**
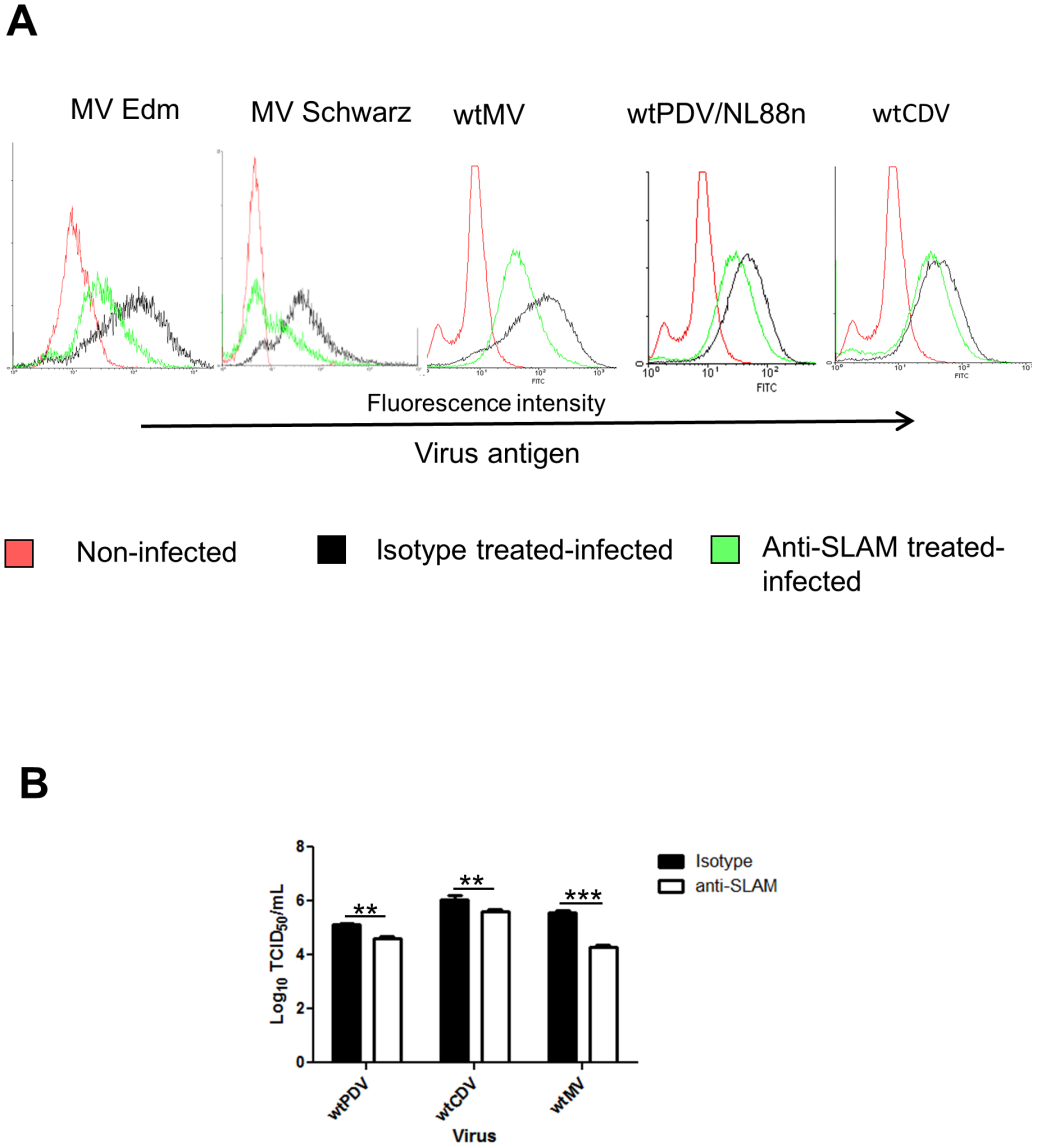
Infection of B95a cells with wtPDV is partially inhibited by anti-SLAM antibody. B95a cells were incubated with anti-SLAM monoclonal antibody or mouse isotype control prior to infection at an MOI of 0.1 with (A) Edmonston MV, Schwarz GFP MV, wtCDV and wtPDV/NL88n at MOI of 0.1 for 2 days followed by fixation and staining (with the exception of Schwarz-GFP MV) with SSPE serum followed by rabbit anti-human FITC and analysed by flow cytometry. (B) Virus was harvested from cells infected with wtMV, wtCDV and wtPDV/NL88n and determination of titres by TCID_50_ in VDS cells. The results are representative of two independent experiments.

Cell fusion but not wtPDV replication was increased in VDS compared to Vero cells. Therefore, the role of SLAM as a cell entry receptor was further examined in B95a cells. Cells were treated with anti-SLAM antibody, infected at an MOI of 0.1 for 2 days with wtPDV/NL88n (previously isolated and grown in B95a cells), Edmonston MV vaccine, Schwarz-GFP vaccine, wtMV and wtCDV strains were used as positive controls. Infected and mock infected cells were fixed, stained with SSPE serum (with the exception of Schwarz-GFP MV) and flow cytometry carried out for all four viruses. All strains of MV are known to use SLAM and infection is inhibited in B95a cells in the presence of anti-SLAM antibody [44]. Our results confirmed this finding. However, the degree of inhibition varied between viruses with Schwarz-GFP MV having the greatest inhibition and wtPDV and wtCDV the least (Figure 3A). In parallel B95a cultures infected with wtMV, wtCDV and PDV/NL88n, virus titres were determined by TCID_50_ (Figure 3B). These results were in agreement with those from flow cytometry and show that wtMV, wtCDV and wtPDV infection was only partially blocked by the anti-SLAM antibody.

### SLAM and PVRL4 expression on CHO cells increases infection of wtPDV

**Figure 4:**
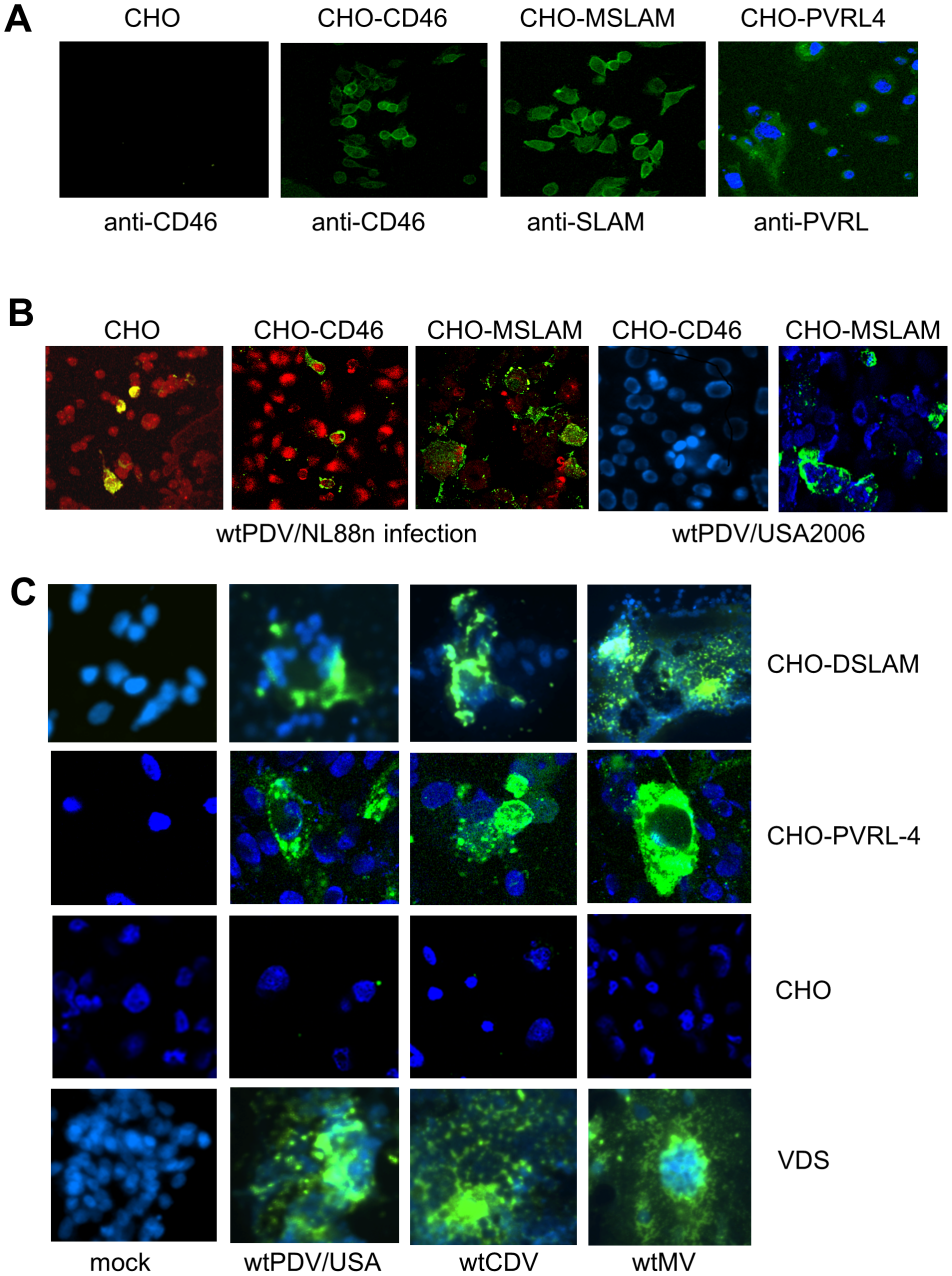
Increased infection of wtPDV infection in CHO cells expressing either SLAM or PVRL4. (A) CHO-CD46, CHO-MSLAM and CHO-PVRL4 cells were stained with their respective receptor antibodies or mouse isotype control, fixed and stained with rabbit anti-mouse FITC. CHO cells were stained in the same manner with anti-CD46 antibody (B) CHO, CHO-CD46, and CHO-MSLAM cells were infected with wtPDV/NL88n and wtCDVUSA2006 (C) CHO, CHO-DSLAM, CHO-PVRL4 and VDS cells with wtPDV/USA2006, wtCDV and wtMV at MOI of 0.1 for 2 days. Cells were, permeabilised, fixed and stained with SSPE serum followed by rabbit anti-human FITC. Nuclei were either stained with propidium iodide and images take in a Leica TCS/NT confocal microscope or stained with DAPI and images taken using a Nikon Eclipse TE2000-U UV microscope (x400).

The results suggest that wtPDV is able to use an alternative receptor to CD46 in Vero cells. We therefore further investigated the use of SLAM as well as the more newly recognised MV and CDV receptor PVRL4 using CHO and receptor expressing CHO cell lines (CD46, PVRL4, MSLAM and DSLAM). CHO cells are not susceptible to infection by MV strains and do not express CD46, SLAM or PVRL4. An alignment using sequences available in GenBank for canine (NP001003084) and phocine (BAH10672) SLAM amino acid sequences show 83% identity overall and 100% at the SLAM binding site [45] validating the use of CHO-DSLAM cells. The expression of CD46, SLAM and PVRL4 was initially confirmed on the respective CHO cell lines by staining, non-permeabilised cells with the appropriate receptor antibody or isotype control. Cultures stained with control antibody showed no staining (not shown) as did CHO cells stained with any of the receptor antibodies, as illustrated for anti-CD46 antibody (Figure 4A). All CHO cell lines and VDS cells (as positive control) were infected for 2 days at an MOI of 0.1 with selected wt viruses. Cells were fixed, permeabilised and incubated with SSPE serum. Only 0 to 2% of cells (in 10 fields of view) expressed antigen in both CHO and CHO-CD46 cells infected with wtPDV/NL88n or wtPDV/USA2006. In contrast the majority of CHO-MSLAM cells were infected throughout the monolayer with these PDV strains (Figure 4B). CHO-DSLAM and, CHO-PVRL4 cells showed infection with wtPDV/USA2006, wtMV and wtCDV (Figure 4C, top and middle panels). However infection was focal in nature compared to VDS cells (Figure 4C, bottom panel) where large syncytia were observed.

**Figure 5:**
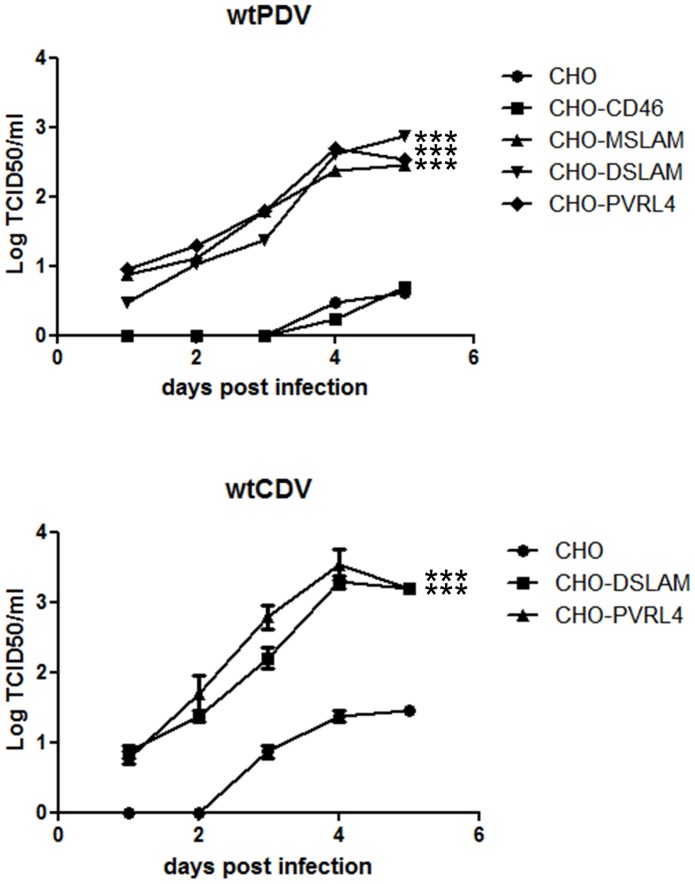
Increased titres of wtPDV are obtained in CHO cells expressing either SLAM or PVRL4. Cells were infected (MOI of 0.1) with wtPDV/USA2006 (all CHO cell lines) and wtCDV (CHO, CHO-DSLAM and CHO-PVRL4). Virus was harvested at 1 to 5 dpi from wtPDV and wtCDV infected cultures and the titre determined by TCID50 in VDS cells. The results are representative of two independent experiments.

Cultures of all CHO cell lines were infected at an MOI of 0.1 for 1 to 5 days with wtPDV/USA2006. CHO, CHO-DSLAM and CHO-PVRL4 cells were also infected with wtCDV. Titres were determined in VDS cells by TCID_50_. Approximately a 2 fold increase in titre was achieved in CHO-MSLAM and/or CHO-DSLAM and CHO-PVRL4 compared to CHO cells for both viruses (Figure 5). However, the titre in these cell lines was 1 to 2 logs lower than those obtained in either VDS or Vero cells (compare Figure 1C and Figure 5) suggesting a restriction in replication and virus maturation in CHO cell lines in general. Overall the results indicate that wtPDV uses both SLAM and PVRL4 as receptors.

### Virus Infection of Vero cells is not inhibited by disruption of GAG chain sulfation or heparinase treatment

**Figure 6:**
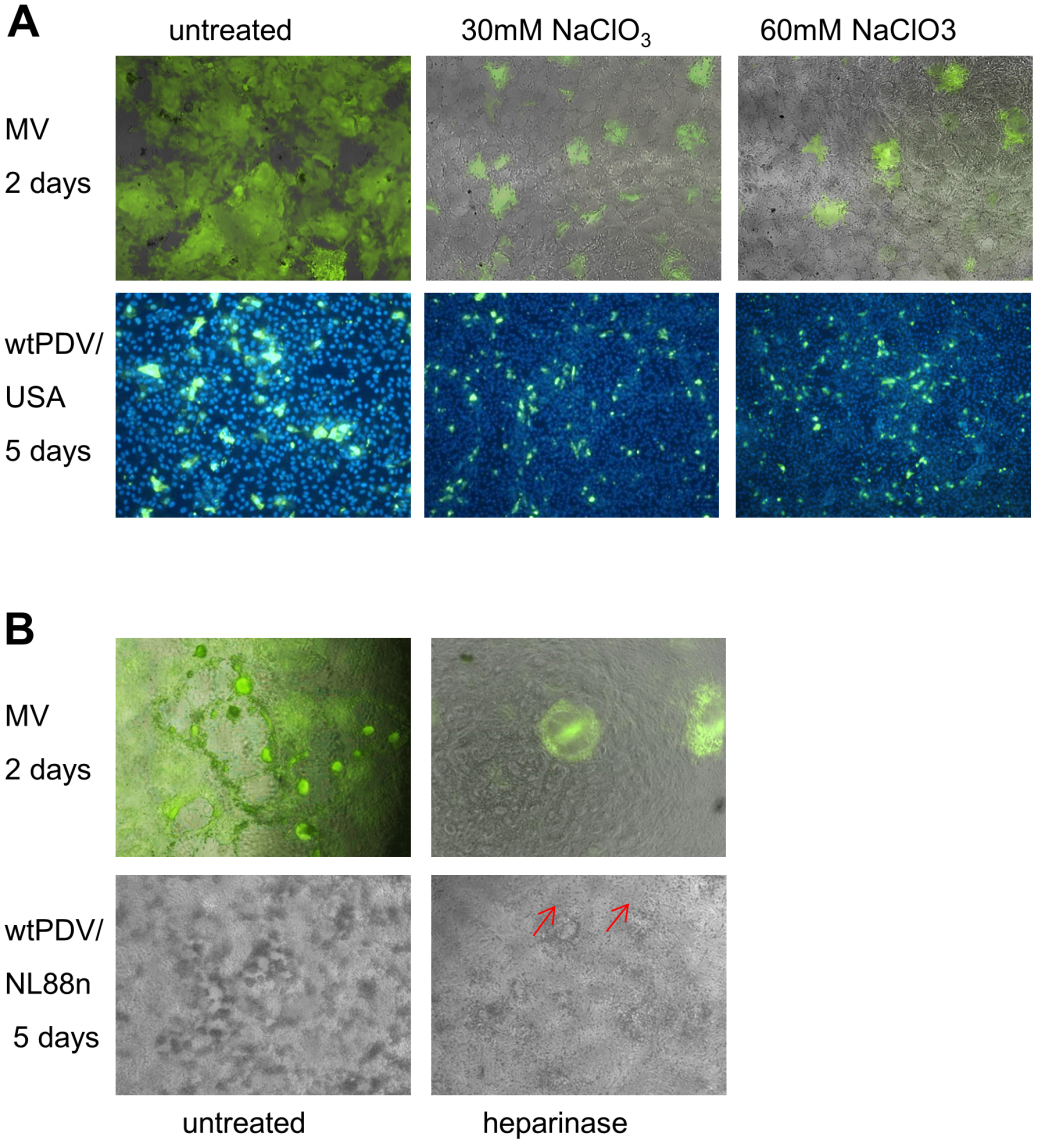
Sodium chlorate and heparinase treatment decreases cell fusion. Vero cells were untreated or treated with 30 mM or 60 mM sodium chlorate for 2 days prior to infection (MOI 0.1) with Schwarz-GFP MV or wtPDV/USA2006. (A) CPE and GFP expression by Schwarz-GFP MV was examined at 2 days by dual phase contrast-UV microscopy (top panel). Cultures of wtPDV were fixed and permeablised at 5 dpi before staining with SSPE antibody and rabbit anti-human FITC (bottom panel). Nuclei were stained with DAPI. (B) Vero cells were treated with 10 U/ml of heparinase for 90 min and infected at an MOI of 0.1 with Schwarz GFP MV or wtPDV/NL88n. CPE was examined at 2 days by dual phase contrast-UV microscopy (top panel) and wtPDV CPE by phase contrast microscopy (bottom panel). Images were taken using a Nikon Eclipse TE2000-U UV microscope (X100).

As we had determined that both wt strains of PDV could infect Vero cells in a CD46 and SLAM independent manner, the receptor usage in these cells was further investigated. Glycosaminoglycans (GAG) have been implicated as a receptor/or to enhance receptor binding of some RPV, CDV and MV strains [15], [39], [40]. We therefore examined the effect on PDV and MV infection of disruption of GAG chain sulfation in Vero cells by using the potent inhibitor of this process, sodium chlorate. Vero cells were treated with either 30 or 60 mM sodium chlorate for 2 days or left untreated prior to infection. Triplicate cultures were infected at an MOI of 0.1 with Schwarz GFP MV or wtPDV/USA2006. CPE was monitored for 2 days for MV and 5 days for wtPDV. Coverslip cultures used for PDV infection were fixed, permeabilised and stained at the end of the experiment for virus as before. MV syncytia in both the 30 mM and 60 mM sodium chlorate treated monolayers were smaller in size compared to control cultures. In wtPDV infected cultures sodium chlorate treatment reduced the limited degree of fusion with this virus further (Figure 6A). Cell free virus was harvested from each well of both MV and wtPDV infected cultures at the end of the sodium chlorate experiment and TCID_50_ titrations carried out. No significant difference in titre was found between untreated and sodium chlorate treated cultures (not shown). Cultures were also treated with 10 U/ml of heparinase for 90 min and infected at an MOI of 0.1 with Schwarz GFP-MV or PDV/NL88n and CPE monitored as before. While, untreated Schwarz GFP-MV infected cultures showed extensive fusion and GFP expression throughout the monolayer, heparinase treated cultures had localised plaques of GFP expression with little fusion. Similarly, untreated wtPDV infected monolayers had extensive rounded cell formation with some fusion while heparinise treated cultures displayed reduced fusion (indicated by red arrows) (Figure 6B). These results indicate that fusion activity rather than virus yield is affected by removal or disruption of GAG.

### Treatment of Vero cells with anti-β1 integrins enhances infection of wtPDV and MV

**Figure 7:**
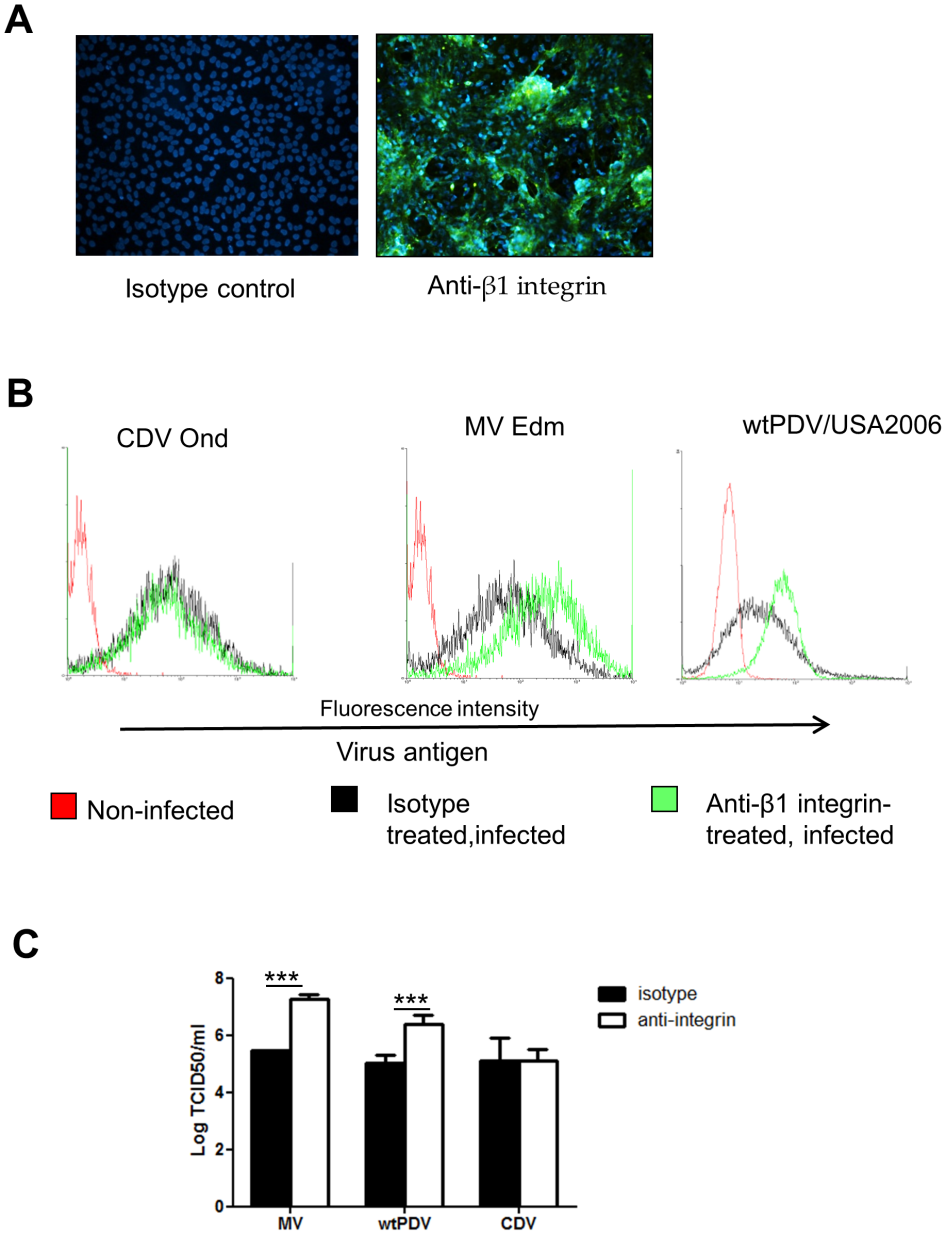
Anti-β1 integrin treatment of Vero cells increases infection of wtPDV and Edmonston MV. (A) Vero cells were examined for β1 integrin expression by staining cells with anti-β1 integrin antibody or mouse isotype control followed by fixation and staining with rabbit anti-mouse FITC. Nuclei were stained with DAPI. Immunofluorescent images were taken using a Nikon Eclipse TE2000-U UV microscope (x100). (B) and (C) Vero cells were incubated with anti-β1 integrin (blocking) antibody or with control mouse isotype, prior to infection (MOI 0.1) for 2 days (Onderstepoort CDV and Edmonston MV) or 5 days (wtPDV/USA2006). (B) Cells were fixed before incubating with SSPE serum followed by staining with rabbit anti-human FITC and analysed by flow cytometry. (C) Virus was harvested and the titre determined by TCID_50_/ml in VDS cells. The results are representative of two independent experiments.

The results indicated use of an additional receptor to SLAM on B95a cells as well as a receptor other than CD46 on Vero cells for wtPDV. We therefore investigated 2 candidate molecules which are in the same tetraspan complex with CD46 and CD9. Most tetraspan molecules co-preciptate with β1 integrins and these have been identified as receptors for a number of viruses [46]. As expected we found that Vero cells showed good expression of β1 integrin (Figure 7A). We therefore examined the effect of anti-β1 integrin blocking antibody on infection of Vero cells with wtPDVUSA/2006, the Edmonston strain of MV and CDV Onderstepoort. Cells were infected at an MOI of 0.1 and stained for virus antigen as before at 2 dpi for MV and 5 days for wtPDV, followed by flow cytometry. Parallel cultures were infected in the same manner and virus titres determined. CDV infection was not affected by anti- β1 integrin treatment. Rather than inhibition there was a marked increase in both Edmonston MV and wtPDV antigen expression (Figure 7B) and infectivity levels (Figure 7C) in anti-β1 integrin treated cells. This suggested that the anti-integrin antibody was enhancing rather than blocking infection of these viruses.

### Treatment of Vero cells with anti HB-EGF antibody reduces infection of PDV

**Figure 8:**
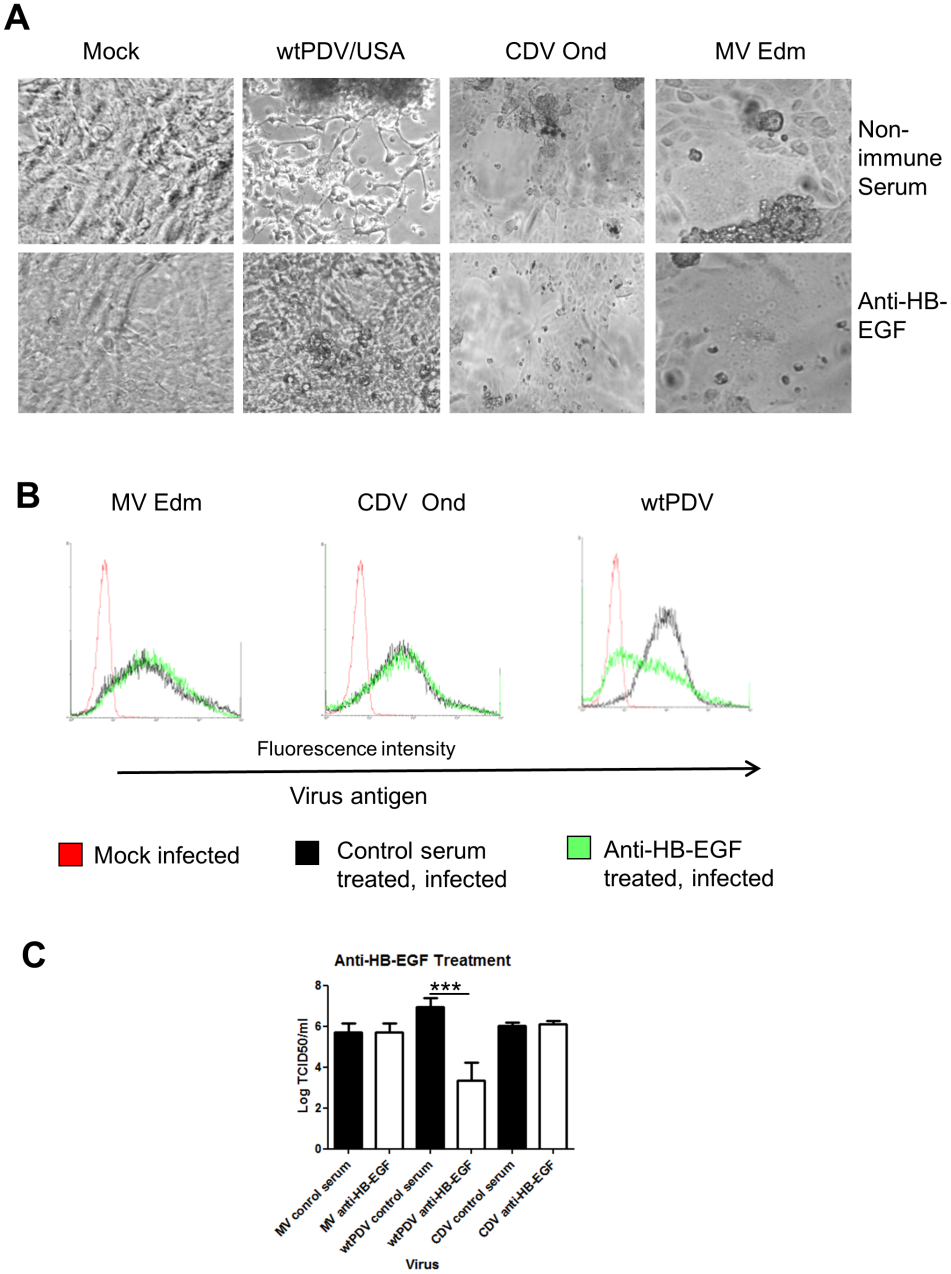
Treatment of Vero cells with anti-HB-EGF reduces infection of wtPDV. (A) and (B) Vero cells were treated with anti-HB-EGF or goat non-immune control serum, prior to infection (MOI 0.1) for 2 days (Edmonston MV and Onderstepoort CDV) or 5 days (wtPDV/USA2006). (A) Cells were examined by phase contrast microscopy and images taken using a Nikon Eclipse TE2000-U UV microscope (x400). (B) Cells were fixed before incubating with SSPE serum followed by staining with rabbit anti-human FITC and analysis by flow cytometry. (C) Virus was harvested from infected cultures and titres determined by TCID50. The results are representative of two independent experiments.

It has been previously shown that proHB-EGF (like CD46) forms a complex with CD9 and integrin alpha3beta1 in Vero cells [34], [35]. We therefore investigated whether this molecule could be a possible receptor for wtPDV/USA. Edmonston MV (which uses CD46) and CDV Onderstepoort (which infects Vero using an unknown receptor) were used for comparison. Cells were treated with anti-HB-EGF antibody or control goat serum, infected at an MOI of 0.1 and examined by phase contrast microscopy at 2 (MV and CDV) or 5 (wtPDV/USA2006) dpi. All cultures treated with control non-immune goat serum showed extensive CPE with all 3 viruses. As expected no inhibition of infection was observed with MV and/or CDV cultures treated with anti-HB-EGF serum. However, in wtPDV infected cultures only a few rounded cell foci were observed at 5 dpi with anti-HB-EGF treatment compared to extensive cell rounding with some fusion in control goat serum treated cultures (Figure 8A). Cultures were fixed, stained for virus antigen as before and flow cytometry carried out. Reduction in virus antigen was very marked for wtPDV but did not occur for MV or CDV (Figure 8B). Virus titres were determined in parallel cultures also infected at an MOI of 0.1. The titre of wtPDV was reduced by approximately 2 logs in anti-HB-EGF treated cultures while MV and CDV were not affected (Figure 8C). This suggests that proHB-EGF may either act as a cell entry receptor for wtPDV or that treatment of cells with the antibody reduces efficiency of virus replication.

### PDV infection and binding is increased in CHO-proHB-EGF cells

**Figure 9:**
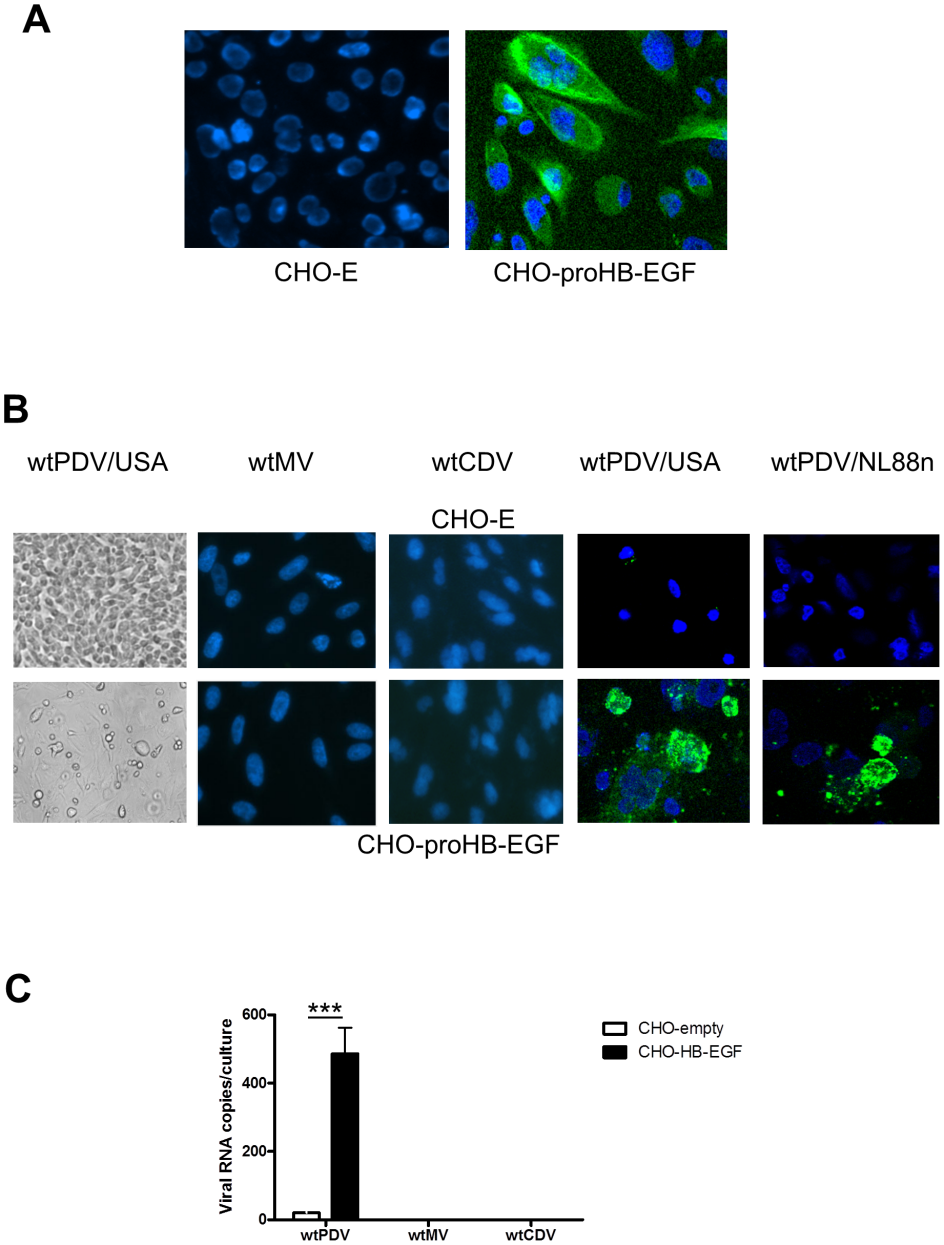
wtPDV infection and binding is increased in CHO-proHB-EGF cells. CHO-empty and CHO-proHB-EGF cells were (A) examined for pro-HB-EGF expression by staining with goat anti-HB-EGF antibody or control goat serum followed by fixation and staining with rabbit anti-goat FITC. (B) Inoculated with wtPDV/USA2006, wtPDV/NL88n, wtCDV or wtMV (MOI 0.1) for 2 days. Cells were viewed by phase contrast microscopy (1st panel) or fixed before staining with SSPE serum and rabbit anti-human FITC (all other panels). Images were taken using a Nikon Eclipse TE2000-U UV microscope (X400). (C) Monolayers were inoculated with wtPDV/USA2006, wtCDV or wtMV (MOI 10) at 4°C for 2 hr. After washing, Sybr green qRT-PCR was carried out and the copy number of virus RNA determined from a standard curve.

To examine further if the membrane anchored form of HB-EGF (proHB-EGF) is used as a receptor for wtPDV infection, a transfected CHO cell line was used. The expression of human proHB-EGF was initially assessed on CHO-empty (CHO-E) and CHO-pro-HB-EGF non-permeabilised cells. CHOE cells carry the control plasmid used to express proHB-EGF and showed no staining as expected while proHB-EGF cells showed expression of the molecule (Figure 9A). CHO-E and CHOpro-HB-EGF cells were inoculated at an MOI of 0.1 with wtPDV/USA2006, wtPDV/NL88n, wtCDV and wtMV (as negative controls) and cultures examined at 2dpi. CHOE cells infected with wtMV and wtCDV (not shown) or wtPDV strains (illustrated for wt PDV/USA2006, Figure 9B, first panel) showed no CPE as did wtMV and wtCDV CHO-pro-HB-EGF inoculated cultures (not shown). However, wtPDV inoculated CHO-proHB-EGF cultures showed cell rounding and areas with limited fusion (illustrated for PDV/USA2006, Figure 9B, first panel). Cells were fixed in paraformaldehyde, permeabilised and stained with anti-SSPE serum and rabbit anti-human FITC. In both CHO-E and CHO-proHB-EGF cells no staining was seen for wtMV or wtCDV (Figure 9B, 2^nd^ and 3rd panesl). However, extensive staining, showing areas of fusion was observed in wtPDV infected cells. In contrast a few cells in CHO-E cultures showed pin point inclusions (Fig 9B, 4^th^ and 5^th^ panels).

We also compared binding at 4°C of wtPDV/USA2006, wtCDV and wtMV to CHO-E and CHO-proHB-EGF cells treated with sodium chlorate to remove heparin (as this might interfere with binding). qRT-PCR was used to determine the relative amounts of virus associated with cell monolayers. In wtMV and wtCDV treated cultures RNA was below the detection limit in both CHO cell lines, while over 20 times more wtPDV RNA copies were detected associated with CHO-proHB-EGF than CHO-E cultures (Figure 9C). Overall these results indicate that proHB-EGF is used as a cell entry receptor by wtPDV.

## Discussion

We have confirmed that wtPDV uses SLAM as a virus receptor and that no major differences in virus titre are found between CHO-MSLAM and CHO-DSLAM cells. It has been determined that only one amino acid change in the H protein of CDV allows the virus to adapt to human SLAM [47]. Furthermore, the SLAM virus H binding site [48] is conserved between canine and phocine SLAM species indicating that titres are likely to be similar in cells expressing the latter. We did not find any significant differences between use of canine and marmoset SLAM by PDV. Tatsuo et al [14] found that MV, CDV and rinderpest virus used their species specific SLAMs more efficiently. This may be dependent on the viruses used and their passage histories.

The sequence of phocine PVRL4 has not been published and is not found in Genbank. However, canine PVRL4 shares 94% identity with the human sequence. Therefore, members of the *carnivore*, i.e. seal and dog sequences would be predicted to have even higher similarity. Furthermore, morbilliviruses have been shown to bind to the V domain of PVRL4 [49] with the virus H protein binding site being highly conserved. This would explain why wtPDV can readily use human PVRL4 and suggests that the virus could also use this molecule in vivo.

Unlike wt MV and wtCDV, wt strains of PDV were found to infect Vero cells with no prior adaptation. In common with RPV and CDV [30], [31] we have demonstrated that wtPDV does not utilise CD46 as a receptor. These results further support the presence of another receptor for wtPDV which we have identified as proHB-EGF on Vero cells. This molecule is also expressed on EBV transformed B cells [50] and may therefore explain why infection of wtPDV was only slightly reduced by anti-SLAM antibody treatment of B95a cells. However, wtCDV infection of B95A cells was also only slightly reduced and may indicate a further unknown receptor(s) on these cells for morbilliviruses.

The number of proHB-EGF molecules on the surface of Vero cells is at least 100-fold less than CD9 molecules [35]. The low density of the receptor may result in relatively reduced virus entry and slower virus spread which would explain why 5 days are required to get good infection levels of wtPDV and limited fusion occurs in Vero compared to VDS cells. wtCDV showed little or no infection of Vero cells and anti HB-EGF antibody did not inhibit infection by the Onderstepoort stain of CDV which infects these cells giving extensive cell fusion after 2 days in a similar manner to vaccine strains of MV. The Onderstepoort virus is therefore likely to use an unknown high density receptor in Vero but it cannot be ruled out that proHB-EGF could also be utilised. ProHB-EGF has been identified as the diphtheria toxin (DT) receptor [35] and although the phocine sequence is unknown other members of the *carnivore* show 89% identity with the human and monkey amino acid sequence. Furthermore, juxtamembrane and transmembrane domains, as well as a proposed heparin-binding region are highly conserved across these species [51] which would explain the lack of adaption required by wtPDV to use the receptor in Vero cells. Although proHB-EGF is expressed in all mammalian species examined to date, species differences in the DT binding site and hence sensitivity to this toxin occur. Vero cells are extremely sensitive to DT whereas mouse and rat cells are resistant. Hamster cells demonstrate intermediate sensitivity [52]. Our results show that wtPDV can bind 20 times more efficiently to Vero than to CHO cells, suggesting that the virus may be binding to the DT binding site but this will require investigation. It has been reported that gut epithelium is extensively infected by PDV in harbour seals [53] whereas in experimental CDV infection of this species the evidence for infection in epithelial is inconclusive [54]. This could be explained by the ability of PDV but not CDV to ustilise phocine proHB-EGF.

Vero cell infection was not inhibited in the presence of an integrin β1 function blocking antibody. In contrast, surprisingly MV and PDV infection was enhanced. Antibody to β1 integrins was previously reported to have no effect on fusion activity of MV in Hela cells. However, viral antigen/infectivity levels were not examined [33]. Antibodies to members of the tetraspans have been found to inhibit or enhance cell fusion depending on the virus, due to either physical separation of the virus fusion machinery from cell-cell contact areas or to inclusion of viral envelope proteins in the tetraspan complex [55]. Furthermore, permissiveness of macrophages to MV using CD46 as a receptor is increased with formation of a complex of CD9, β1 integrins and CD46 [33]. It is therefore possible that anti-β1 integrin treatment is enhancing complex formation in a similar way in the Vero cell membrane allowing closer contact of MV and PDV H and F proteins with CD46 and proHB-EGF, respectively. It will be necessary to examine a range of integrin β1 function blocking antibodies to determine if they increase rather than reduce infection.

ProHB-EGF is also a heparin binding molecule and binding to heparin could enhance infection. Heparinase and sodium chlorate treatments of Vero cells had no effect on released virus titre. However, inhibition of fusion occurred in treated cultures. The effect was less apparent in wtPDV infected cultures due to the more limited level of fusion compared to MV even in untreated cultures. We propose that binding of PDV to heparin or heparin-like molecules associated with proHB-EGF would enhance F protein interaction with the cell membrane but this will require further investigation.

In conclusion, we have confirmed that SLAM is used as a receptor by wtPDV and that the virus does not utilise CD46.The results also indicate that PVRL4 is also used as a receptor in common with MV, CDV and PPRV. This common second receptor may further increase the probability of cross species infection. The finding that wtPDV can use proHB-EGF as a low density receptor in Vero cells indicates that the binding site in the wtPDV H protein requires no or minimal change to utilise this receptor. It remains to be determined if this receptor has a role during PDV infection in the natural host but the lack of adaption required to infect Vero cells and the high conservation of the transmembrane sequence suggests that this is likely to be the case.

## Acknowledgments

We thank Barney o’Loughlin and Samantha Scullion for their excellent technical assistance.
